# Oxidation Behavior of Inconel 740H Nickel Superalloy in Steam Atmosphere at 750 °C

**DOI:** 10.3390/ma14164536

**Published:** 2021-08-12

**Authors:** Barbara Kościelniak, Bartosz Chmiela, Maria Sozańska, Radosław Swadźba, Marcin Drajewicz

**Affiliations:** 1 Department of Materials Science, Faculty of Mechanical Engineering and Aeronautics, Rzeszow University of Technology, 35-036 Rzeszów, Poland; drajewic@prz.edu.pl; 2 Department of Materials Technologies, Faculty of Materials Engineering, Silesian University of Technology, 40-019 Katowice, Poland; bartosz.chmiela@pols.pl (B.C.); maria.sozanska@polsl.pl (M.S.); 3 Łukasiewicz Research Network—Institute for Ferrous Metallurgy, 44-100 Gliwice, Poland; radoslaw.swadzba@imz.lukasiewicz.gov.pl

**Keywords:** A-USC boilers, nickel-based superalloy, high-temperature oxidation, internal oxidation zone, microstructure changes, steam

## Abstract

The oxidation behavior of the nickel superalloy Inconel 740H was studied at 750 °C for 100, 250, 500, 1000, and 2000 h in a steam atmosphere. Microstructure observations were performed using scanning electron microscopes and scanning-transmission electron microscope. The phase identification of existing oxidation products was conducted by electron diffraction in transmission electron microscope. The obtained results showed that the microstructure of Inconel 740H was stable during the oxidation process. The kinetic data showed that the superalloy has the ability to form protective oxide layers that are characterized by good adhesion and no tendency to spallation during the test. The oxidation products were mainly composed of external and internal oxides mainly at grain boundaries. The oxides in the external layer were Cr_2_O_3_, MnTiO_3,_, and α-Al_2_O_3_ after 2000 h of oxidation. Internal oxides were α-Al_2_O_3_ and TiO_2_. The occurrence of discontinuities in the internal oxidation zone was also observed after 500 h of test. It was found that the thickness of the internal oxidation zone was greater than the thickness of the external oxide layer, which proves the strong tendency of the superalloy to form internal oxides after oxidation in the steam atmosphere.

## 1. Introduction

The electric power industry is a strategic sector for every country that ensures security and economic development. Therefore, it is important to improve the technical and economic indicators of boilers in power plants, where special emphasis is placed on their efficiency. The construction of modern units for advanced ultra-supercritical (A-USC) steam parameters with high efficiency (above 46%) and low emission of pollutants into the atmosphere is directly related to the use of modern heat-resistant materials that must meet high quality and utility requirements. The most important requirements of that kind include the high stress-rupture strength, microstructure stability, and corrosion resistance in the environment of exhaust gases, ash, and steam [1,2,3,4].

The high-temperature oxidation resistance of heat-resistant materials in a steam atmosphere is determined by the change in the thickness of the oxides formed on their surface. As the temperature increases, the oxide layer can form faster, which increases its thickness over time. Too large an increase of the oxide layer on the surface of the material can lead to the formation of three potential problems [5,6,7,8]. The first problem is reduction in the thickness of the wall of the superheater and reheater pipes during the oxidation process. The change in the wall thickness may increase internal stresses in the material, which will accelerate the material creep process during boiler operation. Another problem is the increase of the wall temperature of the steam superheater pipes owing to the decrease in the thermal conductivity of the material due to the low thermal conductivity of the oxides’ layer. The increase in wall temperature will affect the acceleration of creep and reduce the corrosion resistance in an exhaust gas. However, the most important problem is the tendency of oxide layers to cracking and spallation due to thermal expansion during boiler operation. The flowing steam can transport the oxidation products to the turbine, wherein the abrasives will cause erosion of turbine blades. Moreover, loose oxides can be deposited inside the pipes, which can reduce the patency of the pipes and, in extreme cases, lead to their blockage [5,6,7,8]. Therefore, it is very important to carefully assess the oxidation resistance of the creep-resistant materials in a steam atmosphere. Evaluation of resistance to oxidation of alloys should not only contain information on the possibility of the formation of oxides on the surface of the alloy, but also determine the kinetics of their formation during oxidation. The kinetics of the alloys’ oxidation in steam depends not only on their chemical composition, but also on other factors, e.g., evaporation of chromium, phase composition of the oxide layer, oxide layer defoliation, grain size of alloy, or internal oxidation [5,9,10]. Therefore, analyzing the oxidation resistance of materials must also consider the various factors that determine the behavior of the material under the influence of steam and temperature, especially in boilers for A-USC parameters. Understanding the behavior of materials allows selecting the right materials to minimize the probability of failure during boiler operation. Out of all the materials suitable for use at critical elements of the A-USC boiler, nickel superalloys exhibit the best corrosion resistance in a steam atmosphere [11,12,13].

Depending on the chemical composition of nickel superalloys and oxidation conditions (temperature, pressure, and corrosive environment), the morphology and phase composition of the oxides formed during their oxidation may vary. However, the oxide layers formed on their surface are thin and have a protective character, regardless of the oxidation state conditions. Nickel superalloys contain 20–25% Cr in their chemical composition. Those materials tend to form a homogeneous layer of Cr_2_O_3_ oxide as a result of the oxidation, which may contain a small amount of Ti, Mn, and Si. In some cases, complex oxides MnCr_2_O_4_, NiCr_2_O_4,_ or MnTiO_3_ may form at the interface between the steam and Cr_2_O_3_ layer. The presence of these oxides prevents the formation of volatile chromium (VI) dihydroxy oxide CrO_2_ (OH)_2_, which causes the evaporation of Cr [14,15,16].

A characteristic feature of some nickel superalloys is their tendency to internal oxidation, regardless of the environment used. As a result of internal oxidation, internal oxides α-Al_2_O_3_ and TiO_2_ are formed in the surface layer of superalloys. The morphology and the penetration depth of the internal oxidation zone depend primarily on the chemical composition of the superalloy, especially on the concentration of Al and Ti. Internal oxides may exist as single precipitates or form clusters of precipitates, mainly at the grain boundaries [14,15,16,17].

In the case of advanced technologies of A-USC boilers (700–760 °C, ca. 35 MPa), the materials that meet these requirements to the greatest extent are Haynes 282, Inconel 617/617 B, and Inconel 740/740H. Accordingly, the most dedicated for use on the thin-walled superheater pipes, precipitation-hardened superalloy Inconel 740H, has the highest rupture strength: 210 and 120 MPa at 700 and 750 °C, respectively, for 100,000 h. [11,12,13].

The superalloy Inconel 740H was selected from precipitation-hardened nickel superalloys, which were created by modifying the chemical composition of the Inconel 740 alloy. The alloy modification consisted of improving the relationship between the concentration of Al, Ti, and Nb and reducing the content of B and Si. Inconel 740H has a high strength property, resulting from solid-solution strengthening of Cr and Co and matrix strengthening by carbides and the precipitation of γ′ phase. Due to the high content of Cr, the superalloy also shows excellent resistance to high-temperature corrosion. Among the available materials, Inconel 740H has the highest creep resistance, which is recommended for use in high-performance pressure components of A-USC boilers [3,18,19,20].

The aim of the study was to characterize the microstructure of the Inconel 740H superalloy and evaluate the oxidation products formed during the oxidation of this alloy in a steam atmosphere at a temperature of 750 °C.

## 2. Materials and Methods

In this study, the investigated material was a straight piece of thin-walled seamless steam superheater pipe (ϕ 31.8 × 6.3 mm), made from Inconel 740H nickel-base superalloy, delivered by Special Metals Corporation. The reheat pipe was supersaturated at 1150 °C and water-quenched. The chemical composition of the investigated superalloy is shown in Table 1.

**Figure 1:**
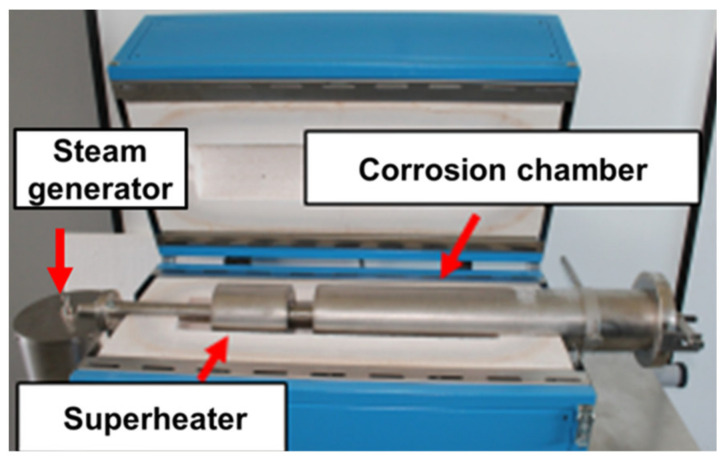
Corrosion furnace for isothermal oxidation test in steam atmosphere under atmospheric pressure.

The isothermal oxidation test was carried out at 750 °C in a steam atmosphere under atmospheric pressure for different times: 100, 250, 500, 1000, and 2000 h in the corrosion furnace (Czylok Company, Jastrzębie-Zdrój, Poland) (Figure 1). The furnace was built of a steam generator and tubular reactor divided into two zones: corrosion chamber and steam superheater (Figure 1). A steam generator was installed on the front panel of the furnace with a capacity of 200 mL per hour. During the tests, steam was continuously supplied to the superheater and then to the corrosion chamber. Distilled water with a conductivity of 0.5 µS/cm was used to generate steam. Samples of 10 × 10 × 6.3 mm were cut from the superheater pipe, ground up to 1200-grit roughness, and then cleaned in an ultrasonic cleaner for 5 min in acetone. Due to the difficulties arising from the possibility of preparing the sample surface for the oxidation test, only the outer surface and the side walls of the samples were ground. The kinetic of oxidation was determined by mass gain. The mass gains for each temperature were measured using an electro balance (±0.1 mg accuracy) after the specimens fully cooled to room temperature.

The surface and cross sections of oxidized samples were observed by using a Hitachi S-3400N and Hitachi S-4200 scanning electron microscopes (SEM, Tokyo, Japan). The chemical composition of oxides was analyzed with X-ray energy dispersion spectrometer (EDS) from Thermo Noran (System Six) (Waltham, MA, USA). The spectrometer was connected to the Hitachi S-3400N microscope. The beam accelerating voltage during SEM observation was 15 kV.

For a deeper investigation of oxidation products, including the chemical and phase analysis, a scanning-transmission electron microscope FEI TITAN 80–300 (STEM) (Hillsboro, OR, USA) equipped with X-ray energy dispersion spectrometer by EDAX was used. For STEM analysis, thin foils were prepared by a focused ion beam (FIB) method with FEI Quanta 200i Dual Beam. The thin foils were cut in the middle of the oxide layer, perpendicularly to the surface of sample. The beam accelerating voltage during STEM analysis was 300 kV.

An electron backscatter diffraction (EBSD) analysis of structural defects and changes in crystallographic orientation in the top layer of the alloy were carried out using an EBSD INCA HKL detector (HKL Technology, Hobro, Denmark) and the Nordlys II analysis system (Channel 5), which was equipped with the Hitachi S-3400N microscope.

## 3. Results and Discussion

### 3.1. Microstructure of Inconel 740H Superalloy

**Figure 2:**
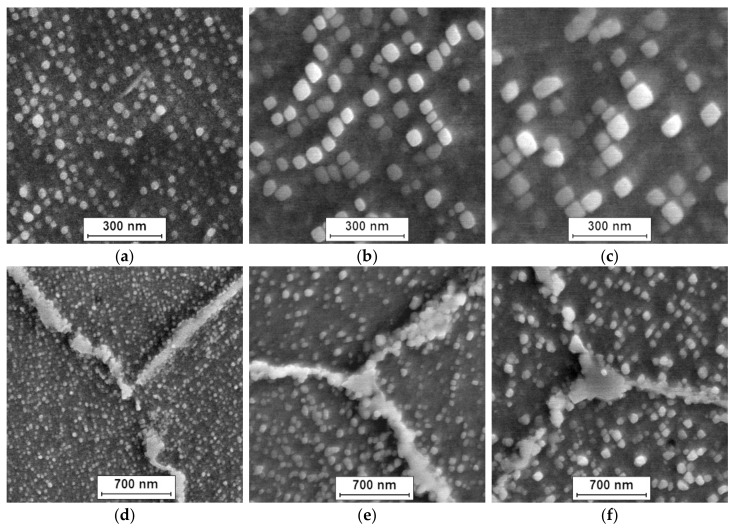
Morphology of γ′ phase (**a**–**c**) and Cr_23_C_6_ carbides (**d**–**f**) in Inconel 740H at various times of oxidation at 750 °C. SEM: (**a**,**d**) 100 h; (**b**,**e**) 1000 h; and (**c**,**f**) 2000 h.

The microstructure of Inconel 740H after oxidation at 750 °C in different times (100, 1000, and 2000 h) is shown in Figure 2.

**Figure 3:**
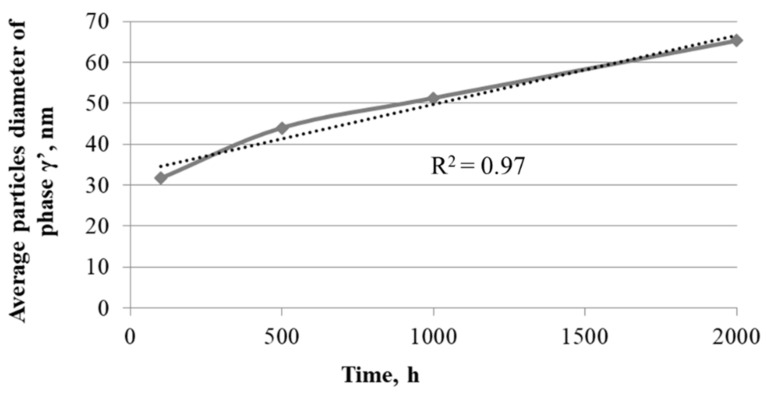
Effect of the heating time during oxidation at 750 °C on value of average particles’ diameter of γ′ phase.

The investigated nickel superalloy had austenitic matrix with γ′ phase inside grains and Cr_23_C_6_ carbides precipitated mainly at grain boundaries and twins. With the increase in the oxidation time, the growth of γ′ phase precipitates and carbides was observed. The Cr_23_C_6_ carbides were characterized by an irregular shape and formed chains along the boundaries of the alloy grains. Based on SEM observation of the alloy after different times of oxidation, it was found that the precipitations of carbides increased and coagulated. Moreover, changes of shape of γ′ phase precipitates from spherical to cubic were also observed. The graph of influence of the heating time during oxidation on value of average particles’ diameter of γ′ precipitates revealed that the relationship between the growth of particles of γ′ phase and the heating time at 750 °C was linear (Figure 3). The correlation coefficients for the size changes of γ′ phase was R^2^ = 0.97.

The obtained results indicated that the change of size of γ′ particles was the result of controlled diffusion processes of alloying elements. Changes in the morphology of γ′ phase precipitations resulted from its chemical composition, especially from the concentration of Al, Ti, and Nb. In particular, the addition of Nb element strongly increased the mismatch of the γ-γ′ network and, thus, the coherent stress at the interface [21]. Despite changes in morphology of γ′ phase, its particles were characterized by high stability at high temperature. Based on the obtained results, it was suggested that the Inconel 740H superalloy will have good creep resistance at a temperature of 750 °C. Good strength properties of alloy result from its precipitation-hardening γ′ phase and Cr_23_C_6_ carbides. Microscopic examination showed the stability of these phases at a temperature of 750 °C, which provided good creep resistance of the alloy. Similar results for the Inconel 740H alloy were obtained by other authors [18,20].

### 3.2. Oxidation Kinetic

**Figure 4:**
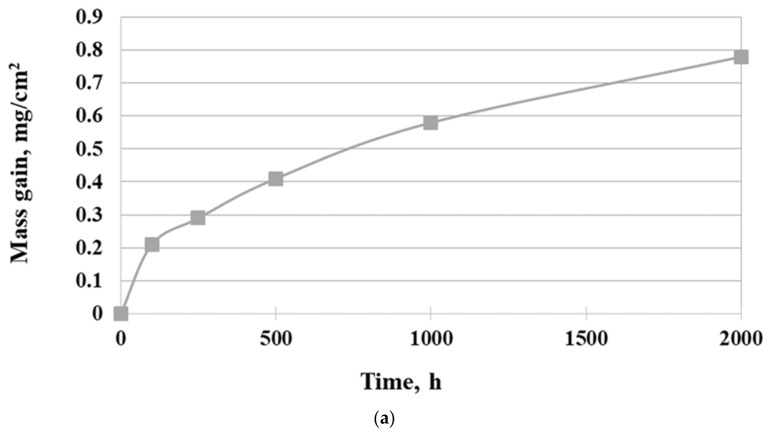

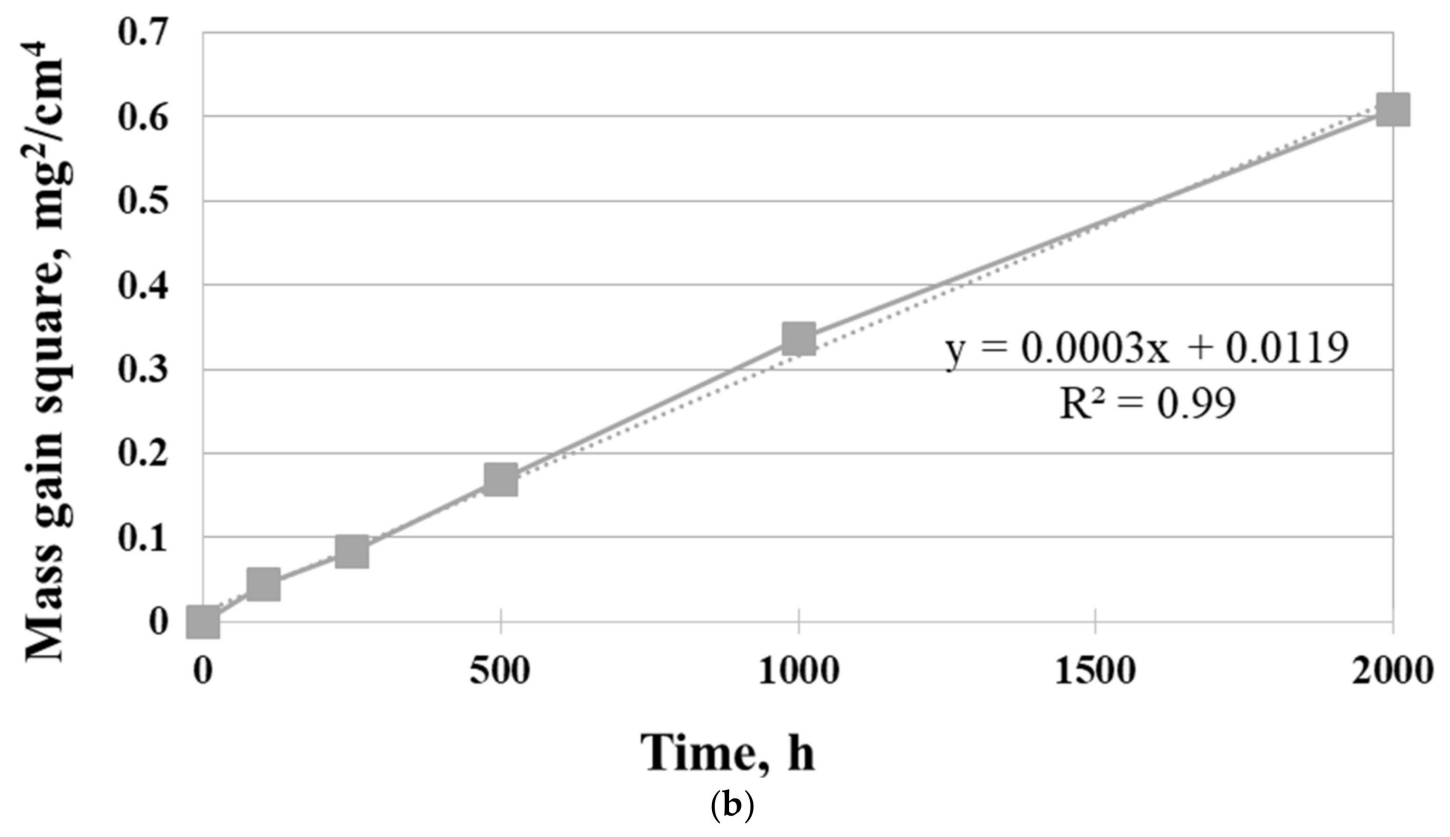
Oxidation behavior of Inconel 740H alloy in a steam atmosphere under atmospheric pressure at 750 °C: (**a**) mass gain and (**b**) square of the mass gain.

The isothermal oxidation kinetic of Inconel 740H superalloy in a steam atmosphere at 750 °C is shown in Figure 4.

As can be seen, the mass gain of superalloy was in accord with the parabolic law. Additionally, the mass loss of samples was not observed. Fitting the curve of oxidation kinetic according to the Formula (1):

(1) $${\left(\frac{\Delta m}{S}\right)}^{2}=k_{p}t+C$$

where Δm is mass gain [mg], S is surface [cm^2^], t is time [h], and k_p_ is parabolic oxidation rate constant [mg^−2^·cm^−4^·h^−1^], the values for oxidation rate constant were 3 × 10^−4^ mg^−2^·cm^−4^·h^−1^. The slope of the linear best fit from the parabolic weight gain plot was the parabolic oxidation rate constant k_p_. Therefore, the parabolic curve indicated diffusion-controlled oxidation behavior of Inconel 740H alloy. The results showed that the investigated superalloy had the ability to form a protective oxide layer, which was characterized by good adhesion to the surface and no tendency to spallation during oxidation in a steam atmosphere. In addition, the extensive evaporation process of Cr during oxidation was not found.

### 3.3. Characteristics of External Oxide Layers Formed on the Surface of Inconel 740H Superalloy

**Figure 5:**
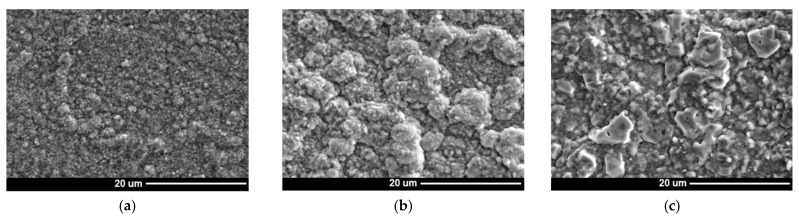

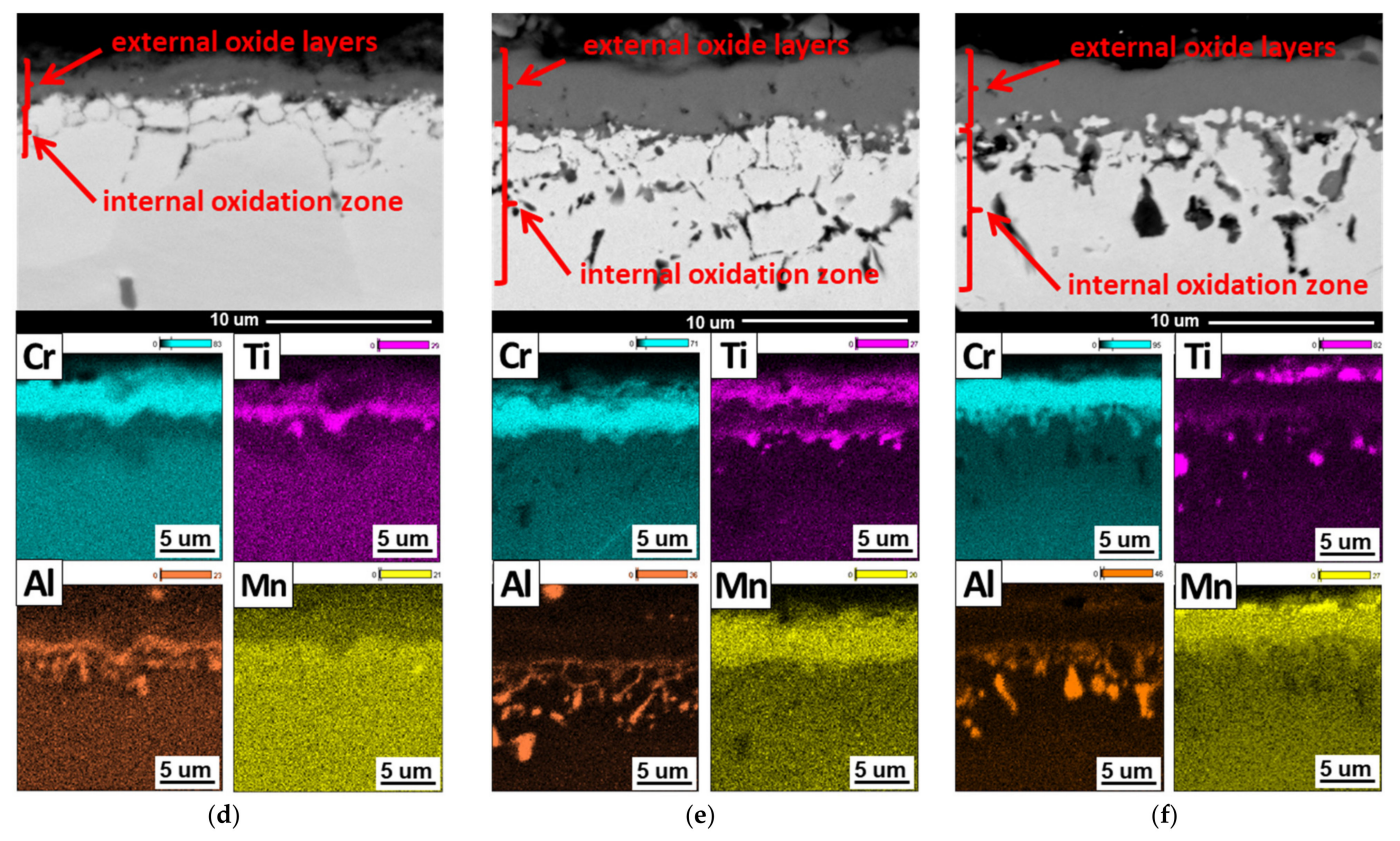
Microstructure of surface (**a**–**c**) and chemical composition of oxides (**d**–**f**) formed during steam oxidation at 750 °C. SEM and EDS: (**a**,**d**) 100 h; (**b**,**e**) 1000 h; and (**c**,**f**) 2000 h.

**Figure 6:**
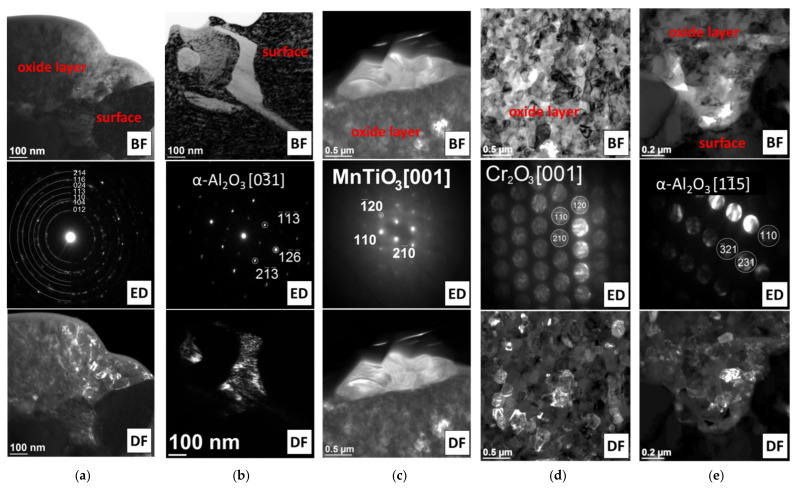
Phase identification of oxides formed during oxidation of Inconel 740H alloy in a steam atmosphere at 750 °C. TEM: (**a**,**b**) 100 h and (**c**–**e**) 2000 h.

**Figure 7:**
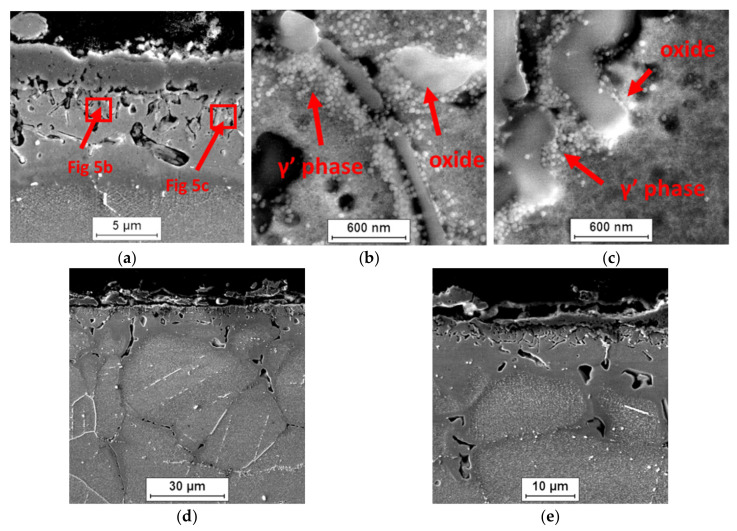
Influence of internal oxidation on changes in the surface layer of Inconel 740H alloy in a steam atmosphere at 750 °C. SEM: (**a**–**c**) 500 h and (**d**,**e**) 2000 h.

The SEM and TEM analyses of the oxide layer formed during steam oxidation in different times showed differences in the morphology of oxides and their chemical and phase composition (Figure 5, Figure 6 and Figure 7). After 100 h of test, small Cr_2_O_3_ and TiO_2_ oxide crystallites were observed on the surface. Increased content of Ti along the grain boundaries of the superalloy showed the presence of TiO_2_ oxides at these areas (Figure 5).

Literature data [22,23] suggest that TiO_2_ oxides form at the beginning of oxidation (before Cr_2_O_3_ oxide). However, the higher Cr content in the chemical composition of the superalloy caused significant growth and dominance of Cr_2_O_3_ oxide on the superalloy surface. In addition, the presence of α-Al_2_O_3_ oxides, which are internal oxidation products, was identified only on the border with the substrate (Figure 5 and Figure 6). The absence of α-Al_2_O_3_ oxides in the oxide layer and the steam-oxide layer interface resulted from the high partial pressure of oxygen (pO_2_) in these areas, which prevented its formation [9]. Based on the obtained results, it was found that the oxide layer after 100 h of oxidation was formed as a result of the diffusion of Cr and Ti.

Lengthening the oxidation time caused a growth of the external oxide layer and depletion of the alloy’s surface layer, which was mainly controlled by the controlled diffusion of Ti and Cr from the matrix (Figure 5). The investigations of the superalloy surface after 1000 h showed that the outer part of the external oxide layer was TiO_2_ oxide crystallites, which grew with time (Figure 5d,e). The formation of TiO_2_ at the steam-oxide layer border resulted from the easy diffusion of Ti ions through the Cr_2_O_3_ layer [22,23]. Moreover, evaluation of the chemical analysis of the external oxide layer also revealed diffusion of Mn, whose beginning started earlier, i.e., after 1000 h of oxidation (Figure 5). Increased concentration of Mn was observed in the areas where TiO_2_ was present. The authors of the papers [22,23,24,25] about the evaluation of the oxidation resistance of Inconel 740 and 740H superalloys described the presence of Mn in the chemical composition of the formed oxide layers. It was noted that the depletion of the surface layer of the alloy in Ti and Cr may cause diffusion along the layer of other components, e.g., Mn [14,16]. Moreover, the Mn ions can also diffuse through the Cr_2_O_3_ layer. Subsequent identification of the phase composition (SAED) of the external oxide layer after 2000 h of superalloy oxidation revealed that Mn reacted with TiO_2_ oxide and formed the MnTiO_3_ complex oxide, which was characterized by an irregular shape of crystallites (Figure 6c).

The results obtained from TEM investigation of the superalloy surface after 2000 h of oxidation showed that the external oxide layer was composed mainly of fine-crystalline Cr_2_O_3_ oxides. Probably, the fine-grained structure of Cr_2_O_3_ facilitated the diffusion of Ti and Mn ions, which resulted in the formation of TiO_2_ and MnTiO_3_ crystallites at the steam-oxide layer interface. Moreover, oxides of α-Al_2_O_3_ were formed at the oxide layer–surface interface of the superalloy (Figure 6). In the papers [14,16] that described the oxidation resistance of the IN 617 superalloy in steam at 900 °C, a similar oxide layer formation process with analogous phase composition was identified and described. Furthermore, the SEM observations revealed that the layer formed on the surface of the Inconel 740H alloy was not discontinuous and characterized by good adhesion to the alloy surface. The obtained results proved the good resistance of the alloy to oxidation in steam at a temperature of 750 °C.

### 3.4. Characteristics of Internal Oxidation Zone Formed during Oxidation of Inconel 740H Superalloy

As a result of the interaction of steam with the IN 740H alloy, an internal oxidation zone was formed in the surface layer of the superalloy. The formation of an internal oxidation zone suggests that oxygen diffused through the oxide layer into the alloy matrix, mainly along the grain boundaries. It is also possible that the absorption of oxygen at the beginning of the oxidation process may have contributed to the formation of the internal oxidation zone. The low partial pressure of oxygen (pO_2_) in the inner layer of the alloy caused the formation of α-Al_2_O_3_ and TiO_2_ internal oxides as a result of the reaction between oxygen and alloy components that were present in the γ′ Ni_3_(Al, Ti) phase (Figure 7).

The α-Al_2_O_3_ and TiO_2_ oxides were thermodynamically stable at low-oxygen partial pressure; therefore, they were formed under these conditions, compared with other oxides, e.g., NiO. Similar effects of the interaction of oxygen with the γ′ phase were described in the papers [15,26]. Microscopic examination of the internal oxidation zone after 100 h of oxidation showed the presence of internal oxides in the form of single α-Al_2_O_3_ crystallites along the grain boundaries. The α-Al_2_O_3_ oxides were characterized by irregular or spherical particles.

Increasing the oxidation time to 2000 h resulted in the intensification of the internal oxide precipitation process. In addition to aluminum oxides, the occurrence of internal oxides containing Ti after 500 h of the oxidation process was also observed. Further studies of the superalloy after longer times of oxidation revealed that the internal oxides were present in the form of clusters of α-Al_2_O_3_ and TiO_2_ fine crystallites, where the α-Al_2_O_3_ oxide was the major constituent of the internal oxidation zone. The size of the internal oxides decreased with the distance from the alloy surface. This was due to the decrease in the diffusion flux of oxygen as a result of moving away from the alloy surface [27].

**Figure 8:**
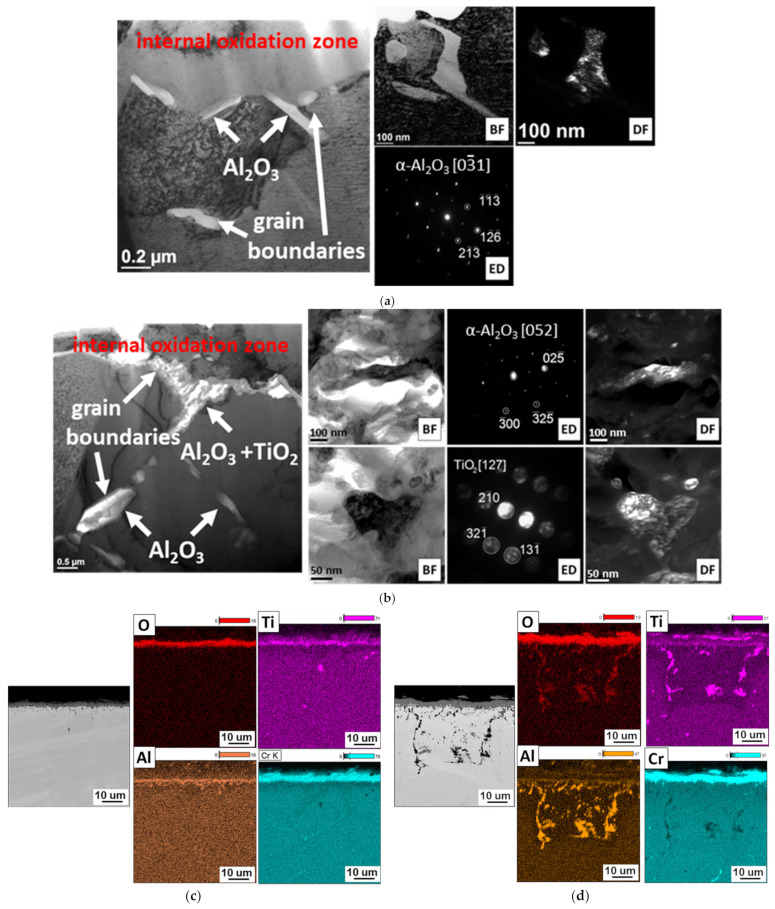
Phase and chemical analyses of internal oxides in Inconel 740H alloy after oxidation in a steam atmosphere at 750 °C. TEM, SEM, and EDS: (**a**,**c**) 100 h and (**b**,**d**) 2000 h.

Transmission electron microscopy observations also revealed that the internal oxides were present not only at the grain boundaries, but also inside them. The obtained results showed that oxygen diffusion also took place through the crystal lattice of the alloy. The authors of [28] found that, in alloys containing more than 1% Al, the formation of α-Al_2_O_3_ due to internal oxidation occurred in the entire grain of the alloy. Based on the results of the chemical composition (EDS) and literature data, it can be concluded that α-Al_2_O_3_ oxides were also formed inside the Inconel 740H grains, while TiO_2_ oxides only formed at the superalloy grain boundaries (Figure 8).

**Figure 9:**
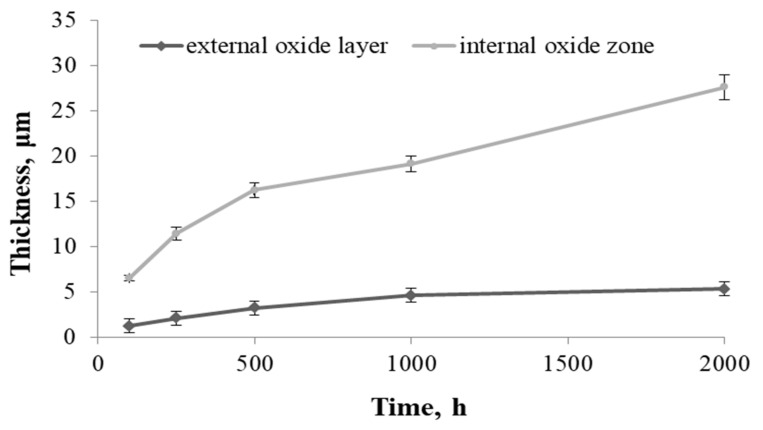
Change in the thickness of the external oxide layer and the internal oxidation zone during the oxidation of the Inconel 740H alloy and in steam at 750 °C.

Examination of the thickness of the external oxide layer and the internal oxidation zone revealed that the internal oxidation process was faster than the formation of the oxide layer on the superalloy surface, which proved the strong tendency of this alloy to form internal oxides (Figure 9).

The formation process of internal oxide continued in a parabolic manner, similar to the growth of external oxide layers. This could mean that the internal oxidation took place through the controlled diffusion of Al, Ti, and O. Therefore, the presence of Al and Ti in the metal matrix highly enhanced the susceptibility to an internal oxidation process at high temperatures in a steam environment. The process was driven by the ability of Ti and Al to oxidation reaction due to high negativity of Gibbs free energy formation [29]. The obtained results suggest that the oxidation kinetics of the nickel superalloy depends not only on the ability to form protective oxide layers, but also on the alloy tendency to form internal oxides [9,26]. The reason for the formation of an increased amount of internal oxides is the presence of the γ′ phase in the matrix of the superalloy (Figure 2). Therefore, the oxidation of the nickel-based superalloy in steam strongly depends on a few factors such as grain size, chemical composition of the superalloy, precipitation processes during oxidation, and the tendency to form an internal oxidation zone.

**Figure 10:**
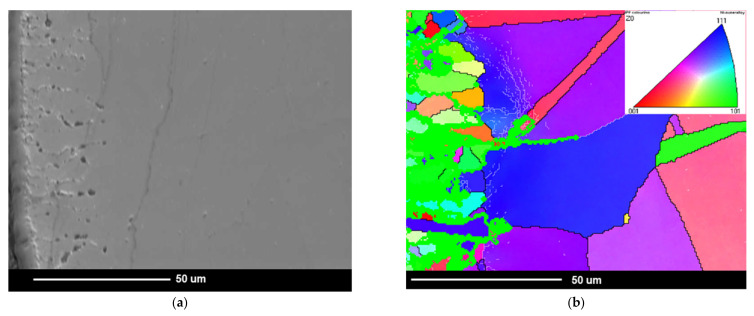
Investigation of grain orientation in Inconel 740H alloy after oxidation for 2000 h at 750 °C in steam. EBSD: (**a**) microstructure of superalloy below the oxide layer, SEM image; (**b**) grain orientation map in the studied area in (**a**), IPF map. Thick lines represent HABs; thin lines represent LABs; color interpretation according to the stereographic triangle where red is <001> direction, green is <101> direction, and blue is <111> direction.

Apart from the presence of the internal oxidation zone, discontinuities (voids) also appeared at the grain boundaries of superalloy. Based on the SEM observations, it was found that the discontinuities appeared after 500 h of oxidation and their amount increased during oxidation (Figure 5 and Figure 7). The paper [24] showed that the discontinuities could have arisen as a result of the Kirkendall effect. The Kirkendall effect consists in the non-equilibrium diffusion of Ti, Cr, Mn, and Al, which diffuse into the oxide layer on the superalloy surface. Apparently, the diffusion of oxygen into the material could also contribute to the formation of grain boundary discontinuities. However, it should be emphasized that the presence of internal oxides improves the corrosion resistance of superalloys. The formation of internal oxides in the alloy matrix improves the adhesion of the oxide layer to the alloy surface by the increase in mechanical bonding. Consequently, the oxide layer shows greater resistance to thermal and mechanical shocks [27]. Moreover, the microstructure of the superalloy below the oxide layer may reduce fatigue resistance, fracture toughness, and tensile strength of the alloy. It is caused not only by the presence of hard oxide particles and the disappearance of the γ′ phase in the internal oxidation zone, but mainly as a result of the internal stresses’ formation in the matrix due to the growth of the internal oxide [30]. This was confirmed by studies on grain orientation of alloy after 2000 h of oxidation in steam by electron backscatter diffraction (EBDS-IPF map) (Figure 10).

Microstructural investigations of Inconel 740H alloy by EBSD technique revealed mostly grains with high-angle boundaries (HABs) with misorientation higher than 15° [23]. After the oxidation test, the low-angle boundaries (LABs) with misorientation lower than 15° were found below, near the internal oxidation zone. In addition, the local change in the crystallographic orientation inside the grains was also revealed. These changes most likely resulted from the formation of internal stresses in the matrix of the alloy. It should also be emphasized that the formation of an internal oxidation zone reduced the wall thickness of the thin-walled steam superheater pipe during oxidation. Therefore, the safe exploitation of the thin-walled steam superheater pipe may be reduced due to the internal oxidation processes involved.

## 4. Conclusions

The results obtained within the present work allowed formulating the following conclusions:

(1) During the steam oxidation of the superalloy at the temperature of 750 °C, there was a change in the size and shape of the γ′ phase precipitates from spherical to cubic as a result of controlled diffusion of elements. There was also coagulation of globular precipitates of M_23_C_6_ carbides and the formation of their chains on the grain boundaries of the alloy. The obtained results showed good stability of the alloy microstructure during annealing for 2000 h at the temperature of 750 °C.
(2) The kinetics of the oxidation of alloys during oxidation in steam was parabolic. Furthermore, the analysis of the kinetics of oxidation of the alloy showed good adhesion of the oxide layer to its surface and no tendency to defoliation.
(3) Qualitative evaluation of the microstructure of the surface layer after superalloy oxidation showed the presence of a fine-crystalline layer of Cr_2_O_3_ and formation of a MnTiO_3_ complex oxide with an irregular shape of crystallites at the steam-oxide layer border. The presence of α-Al_2_O_3_ oxides on the border with the alloy surface was also observed.
(4) The superalloy was characterized by the presence of an internal oxidation zone with internal oxides in the form of a mixture of α-Al_2_O_3_ and TiO_2_ oxides, where the greater amount of internal oxides was α-Al_2_O_3_. The presence of discontinuities in the internal oxidation zone was also observed after 500 h of oxidation, the amount of which increased as a function of time, probably as a result of non-equilibrium diffusion of Ti, Cr, Mn, and Al (Kirkendall effect).
(5) Microscopic examination showed that the thickness of the internal oxidation zone was greater than the thickness of the external oxide layer on the superalloy surface. This proves the strong tendency of Inconel 740H alloy to form internal oxides in the surface layer, which ensures better adhesion of the oxide layer to the substrate and resistance to thermal shocks, which reduces the strength properties of the alloys (presence of hard oxide particles, disappearance of the γ′ phase, and in the internal oxidation zone).
(6) On the basis of studies, it was found that the formation of external oxide layers and the internal oxidation zone was based on diffusion of Cr, Ti, Mn, Al, and O during the interaction of steam. Furthermore, the structural factors that affect the oxidation resistance of the alloy are not only the grain size and chemical composition of the alloy, but most of all precipitation processes—mainly the presence of the γ′ phase. The determining factor influencing the increased amount of internal oxides is the presence of the γ′ phase in superalloy matrix.
(7) The Inconel 740H alloy has a good resistance to oxidation in steam atmosphere at the temperature of 750 °C. However, the presence of the internal oxidation zone reduces the thickness of thin-walled steam superheater pipe and simultaneously reduces the time of its safe use.

## Figures and Tables

**Table 1:** Chemical composition of Inconel 740H superalloy, in wt.% (according to the material certificate provided by the manufacturer).

Cr	Co	Mo	Ti	Al	Fe	Mn	Nb	C	Si	B	Ni
24.50	20.20	0.12	1.32	1.36	2.10	0.50	1.05	0.03	0.16	0.001	Bal.

## Data Availability

The data are available from the corresponding author upon reasonable request.
